# Rayleigh-Wave Dispersion Analysis and Inversion Based on the Rotation

**DOI:** 10.3390/s22030983

**Published:** 2022-01-27

**Authors:** Lixia Sun, Yun Wang, Xinming Qiu

**Affiliations:** “MWMC” Group, School of Geophysics and Information Technology, China University of Geosciences, Beijing 100083, China; slx2018@cugb.edu.cn (L.S.); 3010170010@cugb.edu.cn (X.Q.)

**Keywords:** rotation, translation, Rayleigh wave, dispersion, inversion

## Abstract

Rotational observation is essential for a comprehensive description of the ground motion, and can provide additional wave-field information. With respect to the three typical layered models in shallow engineering geology, under the assumption of linear small deformation, we simulate the 2-dimensional radial, vertical, and rotational components of the wave fields and analyze the different characteristics of Rayleigh wave dispersion recorded for the rotational and translational components. Then, we compare the results of single-component inversion with the results of multi-component joint inversion. It is found that the rotational component has wider spectral bands and more higher modes than the translational components, especially at high frequencies; the rotational component has better anti-interference performance in the noisy data test, and it can improve the inversion accuracy of the shallow shear-wave velocity. The field examples also show the significant advantages of the joint utility of the translational and rotational components, especially when a low-velocity layer exists. Rotational observation shall be beneficial for shallow surface-wave exploration.

## 1. Introduction

Surface-wave exploration is an important method in the field of geophysics used for the detection of the shallow shear-wave velocity structure of the earth, and includes single-station method, two-station method [1], two-plane-wave method [2], ambient noise tomography based on passive source [3], microtremor method [4,5,6], rotational seismic method [7,8], and the most widely used method in the seismic exploration—multi-channel analysis of surface waves (Rayleigh and Love waves) [9,10].

In recent years, numerous experimental studies have used high-speed railway vibration signals for extracting the dispersion curves of the surface waves [11] and used the surface waves detected by an urban telecommunication optic-fiber cable to obtain the shallow velocity structure [12]. Due to the poor applicability of the fundamental-mode surface waves for complex media (containing a low-velocity interlayer and a high-velocity interlayer) [13], the joint utilization of the fundamental and the higher modes has attracted extensive attention [14,15,16,17] and led to better applications [18,19,20,21]. However, the effective identification and accurate extraction of different modes and overcoming the difficulty of misidentification of Rayleigh-wave modes caused by the phenomenon of “mode kissing” has become the main problem faced by the traditional SPAC (spatial autocorrelation) method [22,23,24]. The high-resolution linear Radon transform [25] and the complex vector method, which jointly use the multi-component seismic data, show good results in extracting the surface-wave dispersion curves of different modes [26]. The joint use of the radial and vertical components of seismic translational motions to invert the shallow velocity structure has also been widely used [27,28,29,30,31].

With the development of the rotational seismology, the joint use of translational and rotational components to detect the shallow shear-wave velocity structure has become one of the hotspots in the field of engineering and shallow seismic exploration [32,33]. Six-component (translational- and rotational-component) geophones and seismometers are gradually utilized in these fields [34,35]. However, rotational seismometers’ low sensitivity, large background noise, narrow frequency bandwidths, and high cost still limited their popularization and application [36,37,38]. They are expensive for oil and engineering exploration. There are some methods to obtain the rotational motions indirectly, such as the two-point difference method, which requires the dense seismic array or network [39]. Moreover, wireless seismic geophones make the dense seismic network efficient owing to small logistic and sufficient flexibility [40,41,42]. Therefore, in the near field of strong earthquakes, the rotational motions can be obtained by the wireless seismic geophone resultant.

The comprehensive observation of seismic motions has plenty of advantages on surface wave inversion. The surface-wave phase velocity can be obtained using the rotational rate measured by the rotational seismometers and the acceleration measured by the translational geophone on a single location without an array of geophones [7]. The apparent shear-wave speed, which is defined by the rms (root-mean-square) amplitude ratio of the translational component and rotational component, can be used for single-station local S-wave velocity tomography [32]. Additionally, the energy of surface waves is stronger than that of body waves on the rotational components, which can be used for surface wave inversion [43]. 

However, compared with the translational seismic method, other advantages of the rotational observation are attempted to be clarified in this paper. Aimed at three typical layered models in shallow engineering geology, we analyze the Rayleigh-wave dispersion characteristics of the rotational component differing from the translational components through theoretical simulations. In addition, we compare the single-component inversion with the multi-component joint inversion with the numerical and field data, which are calculated with the two-point difference method by using the translational field seismic data. It is demonstrated that the understanding and utilization of the surface-wave dispersion curves on rotational components is helpful to improve the detection accuracy of the shallow shear-wave velocity structure.

## 2. Theoretical Foundations

### 2.1. Calculation of the Rotational Component

**Figure 1:**
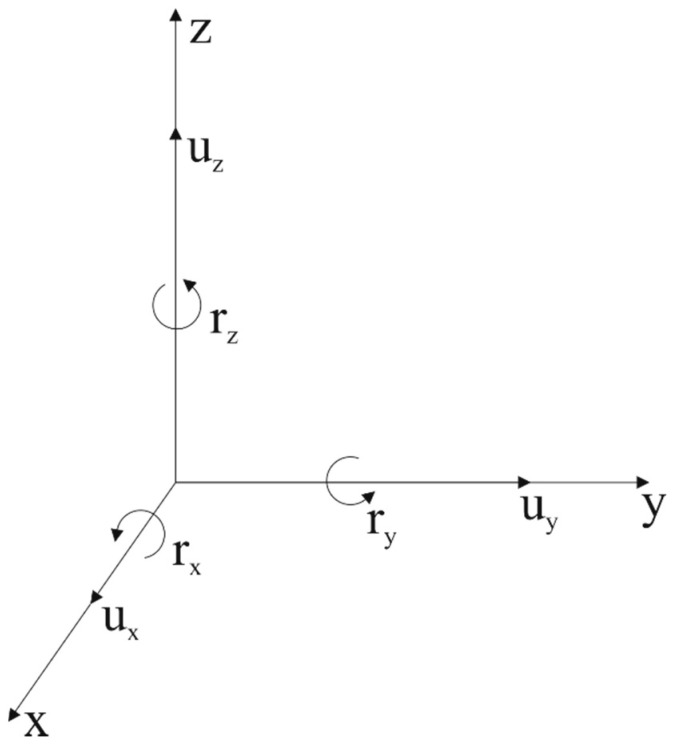
Schematic diagram of the rotational motion.

The motion of a particle includes translation, rotation, and deformation. In space, the motion of an arbitrary point can be expressed as three translational components along the axis and three rotational components around the axis (Figure 1) [44]. In the traditional linear elastodynamics theory, the rotational tensor is defined as follows:

(1) $$\vec{r}=\frac{1}{2}\nabla \times \vec{u}$$

where $\vec{r}$ is the rotational tensor, $\vec{u}$ is the displacement. It is obviously that the rotation tensor is half of the curl of the displacement vector.

In order to completely describe the ground motion, six degrees of freedom in a three dimensional world are required, as shown in Table 1 [43]. In the two-dimensional case, it becomes three components—the radial (u_x_), vertical (u_z_), and yaw (r_y_) components. We analyze the wave-field characteristics of three components under the assumption of two-dimensional linear small deformation and use the surface-wave dispersion curves extracted from different components to predict shear-wave phase velocity.

Since the actual rotational observation is mainly the rotational rate, it is necessary to obtain the rotation rate in the numerical simulation by the derivative of Formula (1) with respect to time (*t*). Further, the rotation rate R_y_ can be obtained through discrete difference calculation by the wave-field velocity, which can be expressed as:

(2) $$R_{y}\left(x,z,t\right)=\frac{1}{2}(\frac{v_{x}\left(x,z+\Delta z,t\right)-v_{x}\left(x,z,t\right)}{\Delta z}-\frac{v_{z}\left(x+\Delta x,z,t\right)-v_{z}\left(x,z,t\right)}{\Delta x})$$

where $R_{y}\left(x,z,t\right)$ is the calculated rotational value, which is used in our simulation analyses, Δx and Δz are 0.2 m, $v_{x}\left(x,z,t\right)$ and $v_{x}\left(x,z+\Delta z,t\right)$ are the radial component velocities at different depths, and $v_{z}\left(x,z,t\right)$ and $v_{z}\left(x+\Delta x,z,t\right)$ are the vertical component velocities at different points.

### 2.2. Rayleigh Wave Simulation 

In this paper, the two translational components of surface wave are synthesized by the convolution of the dispersion curves and eigenfunction (modal summation method) [45] with the CPS (The Computer Programs in Seismology) software [46], because the method is based on the analytical solution of the surface wave and the surface-wave fields are considered as pure. Then, the rotational component wave fields are calculated with translational records according to the Formula (2), although the rotational components can be simulated with the finite difference method [47,48]. While in the real data testing, we use the two-point difference method [49] to obtain the rotational component R_y_, since the rotational seismometers are not popular and there is usually a lack of rotational observation in the field of engineering seismic prospecting.

### 2.3. Method of Surface-Wave Dispersive Energy Imaging and Surface-Wave Inversion 

High-resolution Radon transform is used to calculate surface-wave dispersive energy in this paper. The Radon coefficients are obtained with formula [25]:

(3) $$\left(\lambda I+W_{m}^{-H}L^{H}W_{d}^{H}W_{d}LW_{m}^{-1}\right)\tilde{m}=W_{m}^{-H}L^{H}W_{d}^{H}W_{d}d$$

where $\tilde{m}=W_{m}m$, $W_{d}$, and $W_{m}$ are the weighted matrices, L and $L^{H}$ are the operator matrices, λ is the regularization parameter, and I is the identity matrix. m is the Radon coefficient in the frequency–velocity (f–v) domain, which is a complex number. d is the seismic data in the frequency–offset domain.

The dispersive energy in the f–v domain can be imaged with the module of the Radon coefficients [14]. Then, the imaged energy is normalized at each frequency, which can remove the effect of the source wavelet spectrum [26].

Furthermore, we extract the surface-wave dispersion curves from different components and inverse the shallow underground velocity structure using the Rayleigh wave inversion program of CPS software—the surf96 module. Under the smoothness constraint, a damped least-square inversion method is used to find the most suitable model matching the observed values and invert the velocity structure by the dispersion curves of surface waves.

## 3. The Wave-Field Characteristics of the Typical Shallow Models

Considering the generality of the discussion, we define three typical models based on the common geological structures in shallow engineering geology—horizontal layered model with velocity increasing with depth and layered model containing low-velocity interlayer or containing high-velocity interlayer. The model parameters are shown in Table 2. We simulate the surface-wave fields with a vertical concentrated force source (a 25 Hz Ricker wavelet) at the surface. With the sample interval of 0.5 ms, there are 49 receivers arrayed in line at the surface with 1 m intervals and the nearest offset is 5 m.

**Figure 2:**
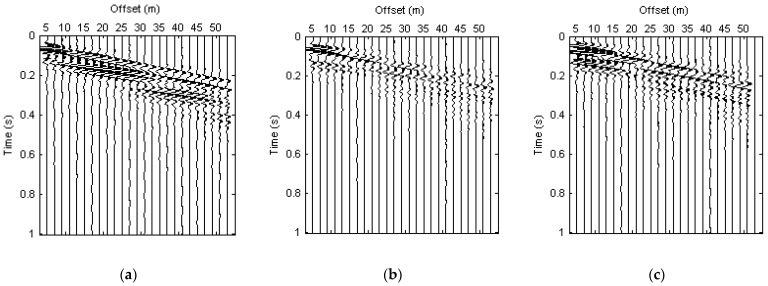
Synthetic data for Model 2. (**a**) X component; (**b**) Z component; (**c**) R_y_ component.

The synthetic data for Model 2 are illustrated in this paper in Figure 2. It can be clearly seen that there is strong Rayleigh wave energy in the shape of a broom on the three components. The energy of Rayleigh waves is much stronger on the vertical and rotational components than that on the radial component.

**Figure 3:**
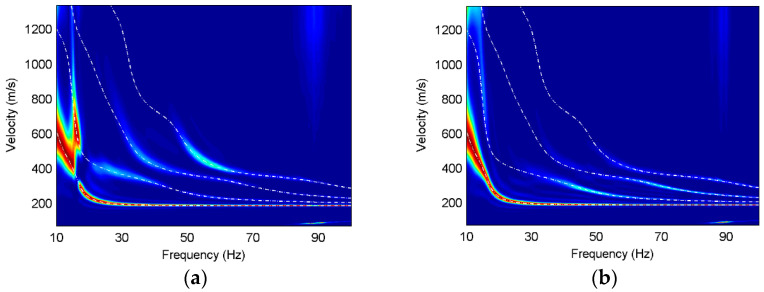

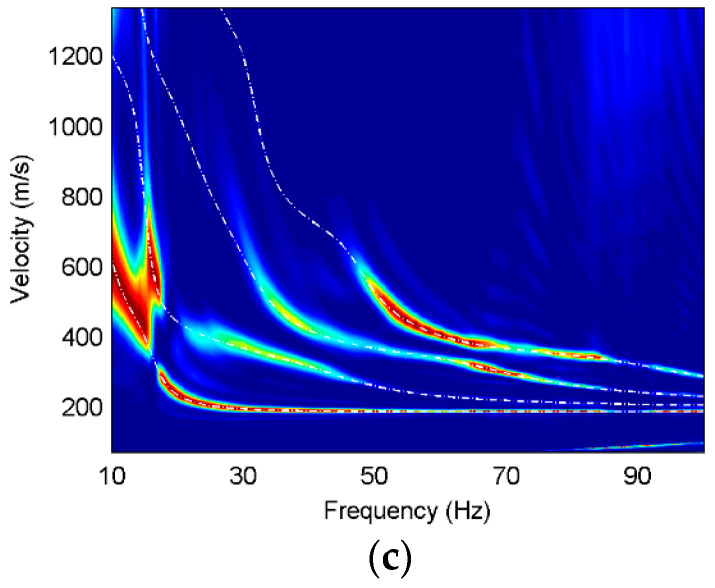
Dispersion images for Model 1, where the white dashed lines are the theoretical dispersion curves and the red color represents the maximum energy. (**a**) X component of Model 1; (**b**) Z component of Model 1; (**c**) R_y_ component of Model 1.

**Figure 4:**
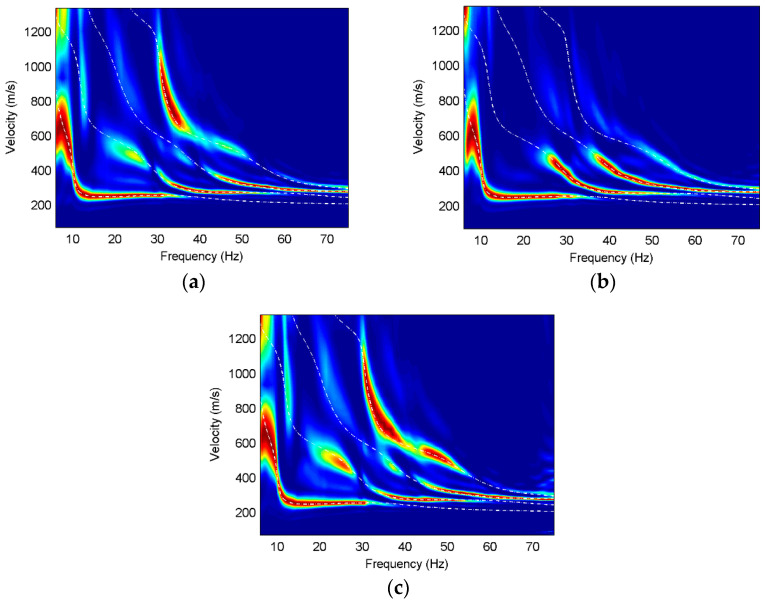
Dispersion images for Model 2, where the white dashed lines are the theoretical dispersion curves and the red color represents the maximum energy. (**a**) X component of Model 2; (**b**) Z component of Model 2; (**c**) R_y_ component of Model 2.

**Figure 5:**
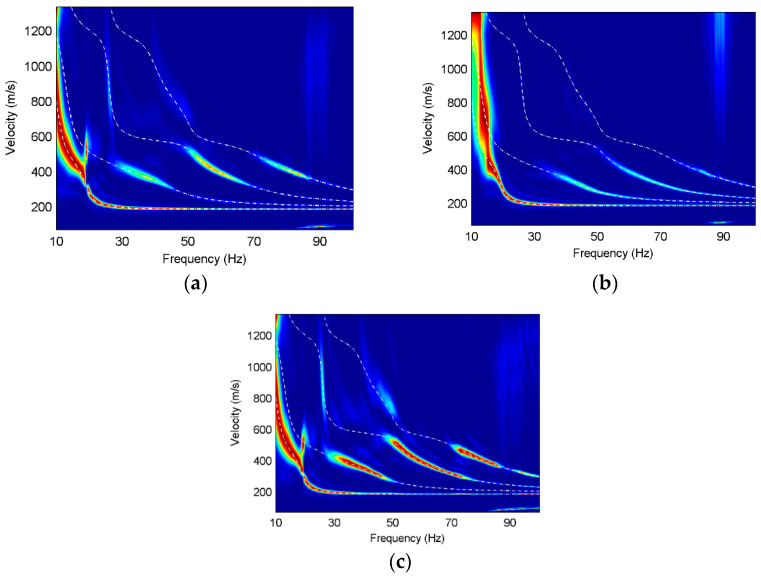
Dispersion images for Model 3, where the white dashed lines are the theoretical dispersion curves and the red color represents the maximum energy. (**a**) X component of Model 3; (**b**) Z component of Model 3; (**c**) R_y_ component of Model 3.

In order to further analyze the Rayleigh wave characteristics on different components, we use high-resolution linear Radon transform [25] to obtain the multi-mode surface-wave dispersive spectra. The dispersions of three components are normalized at each frequency and the theoretical dispersion curves of different modes are calculated for contrast, as shown in Figure 3, Figure 4 and Figure 5.

It can be found that obvious fundamental and higher modes exist on the three components, and the dispersion curves for different models basically correspond well with their theoretical dispersion curves. The fundamental mode of Model 1 is dominant on all components, and the fundamental mode on the vertical component has energy distribution at 15–18 Hz, while that on the other two components is discontinuous at the same frequencies. The first higher mode on the radial and rotational components has comparative amplitude responses at 10–15 Hz frequencies, while not all higher modes are present in the dispersion images. The higher modes on the translational components have much weaker energy than those on the rotational component at 15–100 Hz frequencies and the higher-mode dispersion curves can hardly be extracted from the translational components. In contrast, the higher modes, especially the third higher mode, have strong energy on the rotational component and the dispersion information is relatively complete.

The fundamental modes of Model 2 on three components are similar while the higher modes have different characteristics on different components. The first higher mode and the second higher mode on the vertical component have much stronger energy than those on the other two components at the frequencies of 25–40 Hz, while the third higher mode is almost absent on the vertical component, but dominant on the radial and translational components. The dispersion images on the rotational component are slightly better than those on the radial component of Model 2, which is reflected in the energy of the first higher mode at 20–30 Hz frequencies, the energy of the second higher mode at 32–40 Hz frequencies, and the energy of the third higher mode at 40–52 Hz frequencies. It is obvious that the rotation has wider spectral bands than the translations in the horizontal layered model containing a low-velocity interlayer.

In Model 3, the higher modes on the vertical component have weaker energy than the other components. It can be observed that the dispersive energy of the vertical component at 10–15 Hz frequencies is misidentified as the fundamental mode, which is the phenomenon of mode misidentification. However, mode misidentification can be overcome using multi-component seismic data. The fundamental-mode and higher-mode dispersion curves on the radial and rotational components match well with the theoretical dispersion curves. The energy of the higher modes on the rotational component is much stronger than that on the radial component. Furthermore, the dispersive energy of the higher modes on the rotational component has wider frequency bands and more high-frequency information than that on the radial component.

Therefore, we can deduce that the rotational components are helpful to pick up dispersion curves of different modes, which is beneficial for the surface-wave inversion and geological interpretation.

## 4. Rayleigh Wave Inversion

**Figure 6:**
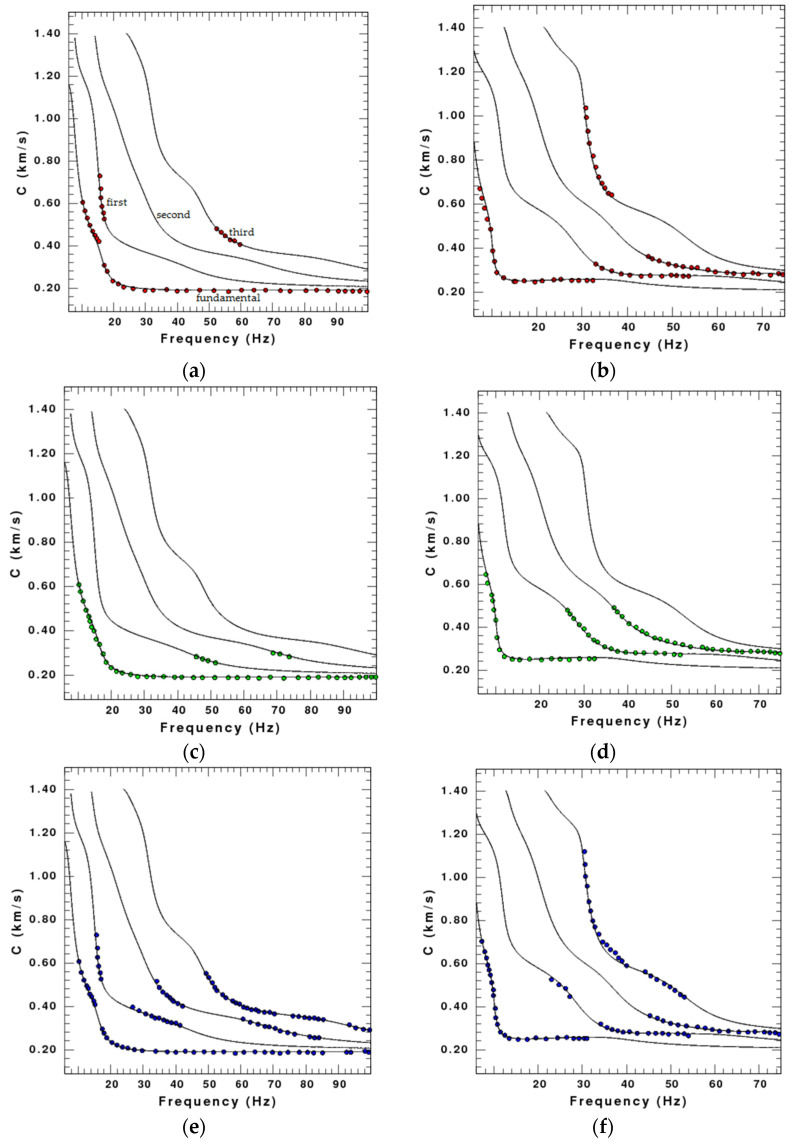
Dispersion curves for Model 1 and Model 2, where the black lines are the theoretical dispersion curves and different shapes of points correspond to the different modes. (**a**) X component of Model 1; (**b**) X component of Model 2; (**c**) Z component of Model 1; (**d**) Z component of Model 2; (**e**) R_y_ component of Model 1; (**f**) R_y_ component of Model 2.

In order to verify the effect of rotations on the Rayleigh wave inversion, we pick up the phase velocities with the maxima energy from the dispersion spectrum and use the damped least-square inversion method to invert the shear-wave velocity of different models. Because the inversion of Model 3 draws the similar conclusion to that of Model 2 in the middle low-velocity layer, we only display the results of Model 1 and Model 2. The dispersion curves of Model 1 and Model 2 used for inversion are shown in Figure 6, and the comparison of bandwidths among radial, vertical, and rotational data is shown in Table 3.

**Figure 7:**
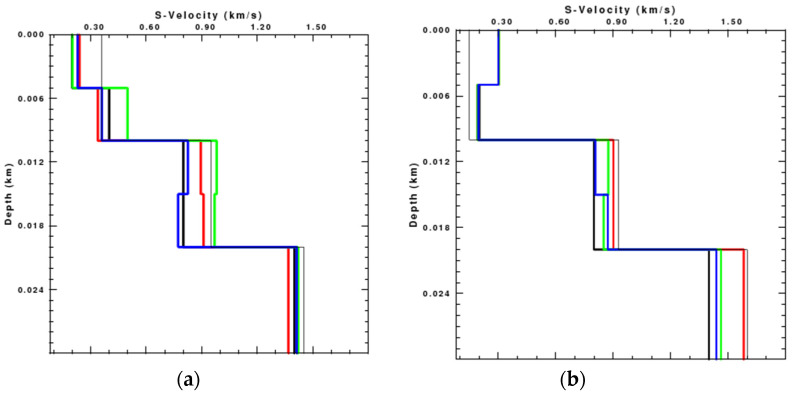
Results of the Rayleigh wave inversion using the radial (red line), vertical (green line), and rotational (blue line) seismic data respectively. The black thick line is the theoretical S-wave velocity, and the black thin line is the initial S-wave velocity. (**a**) Model 1; (**b**) Model 2.

It is obvious that the dispersion curves on the rotational components are more complete than those on the translational components. The results of the inversion, terminated after 20 iterations, are shown in Figure 7. Limited by space of the paper, we only show the single-component inversion results for clearer comparison, since there are great similarities between the rotational component inversion results and the joint multi-component (radial, vertical, and rotational components) inversion results in the numerical test.

It can be found that the results of the inversion using the rotational component are much better than those using translational components, especially in the deep layer. The S-wave velocity of Model 1 inverted by the rotational component is close to the theoretical model at the depth of 0–5 m and 10–20 m, while that inverted by the translational components has a relatively greater deviation from the theoretical model. In addition, the inversion result using the rotational component almost consists of the theoretical S-wave velocity at the depth of 5–10 m and in the deep layer. The S-wave velocity of Model 2 inverted by the three components approximates the theoretical S-wave velocity within 10 m depth, but is quite different in the deep layer. The inversion results using translational components show a much greater velocity in the deep layer while the inversion using rotational component is close to the theoretical model. The comparison demonstrates that the inversion results using the rotational component are more accurate than those using the translational components.

## 5. Noisy Synthetic Data Test

**Figure 8:**
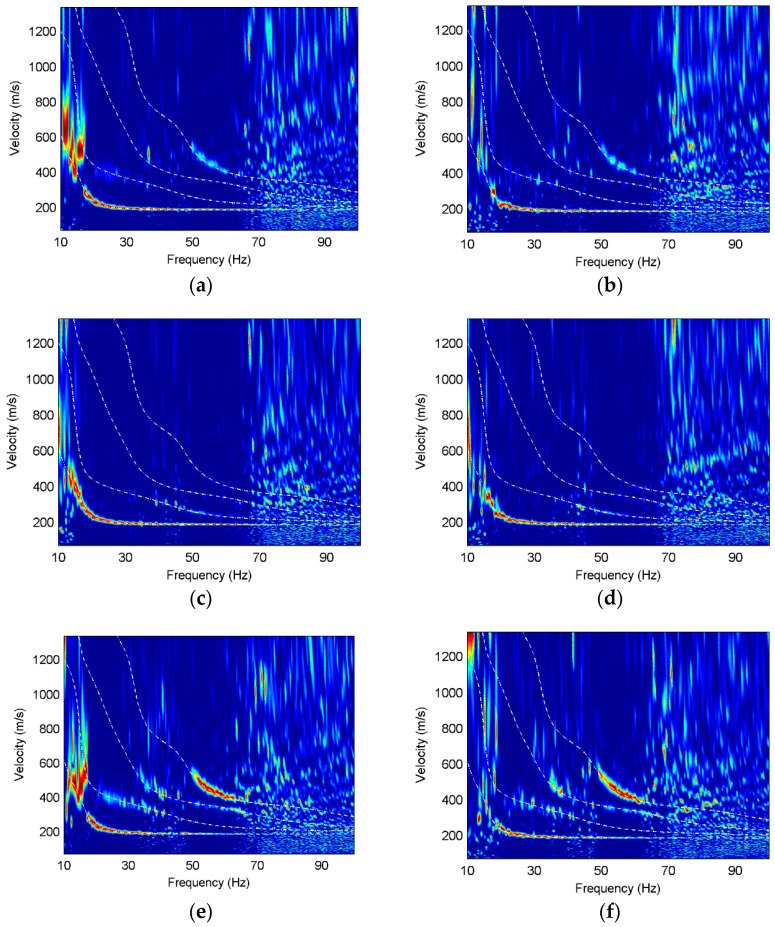
Dispersion images of noisy data of Model 1, where the white dashed lines are the theoretical dispersion curves and the red color represents the maximum energy. SNR of the left column is 2.8 and SNR of the right column is 1.7. (**a**) X component; (**b**) X component; (**c**) Z component; (**d**) Z component; (**e**) R_y_ component; (**f**) R_y_ component.

**Figure 9:**
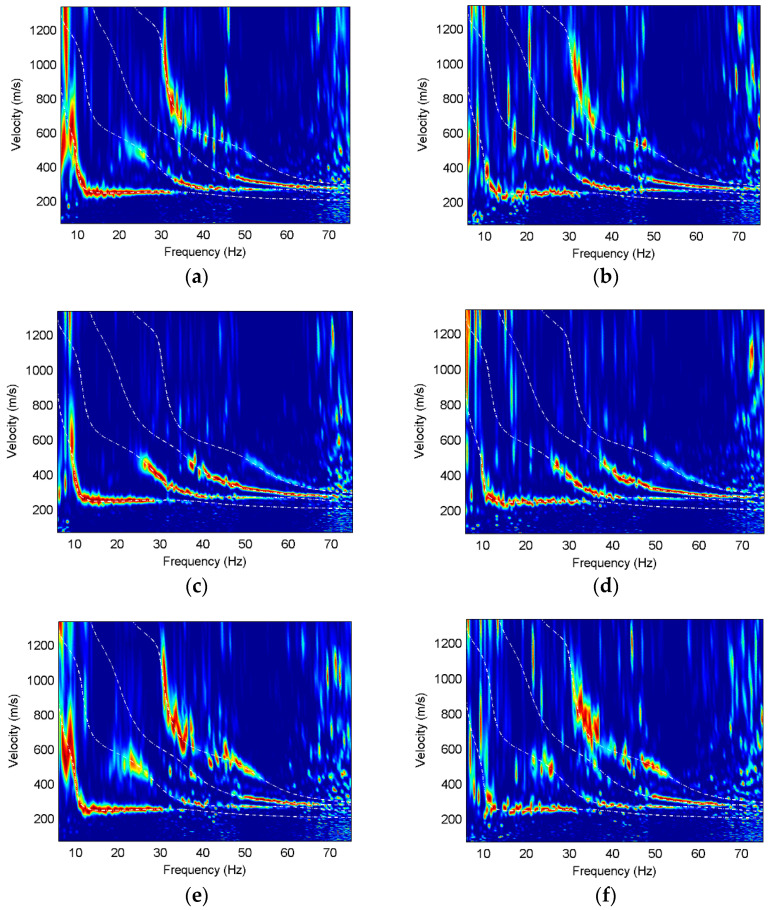
Dispersion images of noisy data of Model 2, where the white dashed lines are the theoretical dispersion curves and the red color represents the maximum energy. SNR of the left column is 2.8 and SNR of the right column is 1.7. (**a**) X component; (**b**) X component; (**c**) Z component; (**d**) Z component; (**e**) R_y_ component; (**f**) R_y_ component.

Considering field applications, we add different level white noises to the seismic data of the Model 1 and Model 2. The signal-to-noise ratio (SNR) is 2.8 and 1.7, respectively. The normalized dispersion images of different components are shown in Figure 8 and Figure 9.

It is obvious that there is only the fundamental-mode energy on the translational component noisy synthetic data of Model 1, and the higher modes are masked by the noise, while the third higher mode is relatively strong on the rotational component, as well as the fundamental mode. Reducing the signal-to-noise ratio has a great influence on the dispersive energy at low frequencies, especially at the frequencies of 10–15 Hz. The fundamental mode on the radial component noisy data of Model 2 is dominant while the higher modes are discontinuous. In contrast, the first and second higher modes on the vertical component have comparative energy and the widest frequency bands since they are less affected by noise. The first higher mode exists at the 20–25 Hz frequencies on the rotational component, but is absent on the translational components. The third higher mode is relatively stronger on the rotational component than that on the other components. This comprehensive comparison illustrates that the Rayleigh waves on the rotational component have stronger anti-noise performance and more complete higher-mode information.

**Figure 10:**
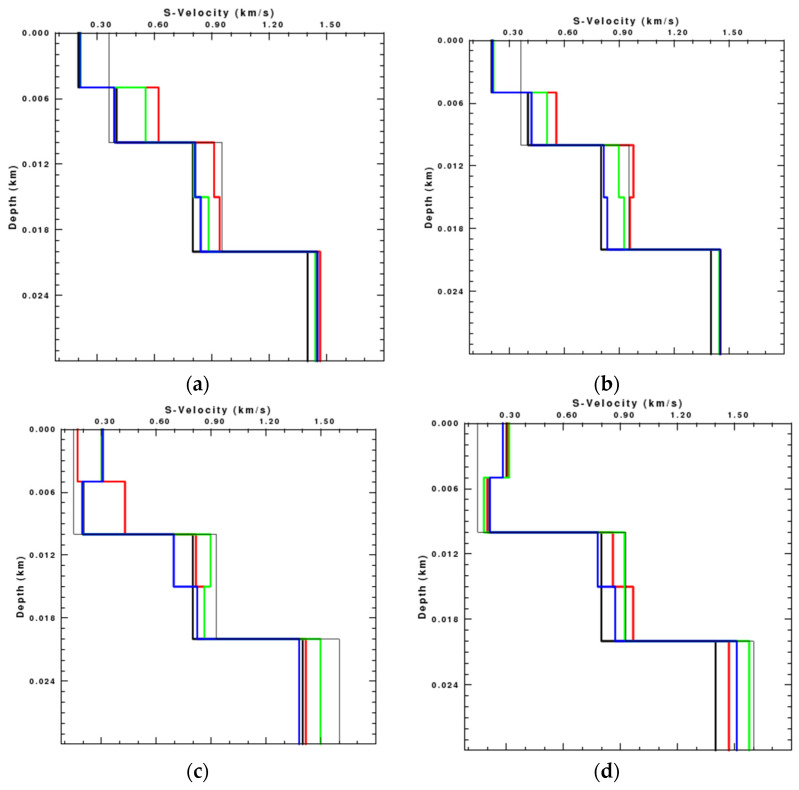
Inversion results of the noisy data using the radial (red line), vertical (green line), and rotational (blue line) components, respectively. The black thick line is the theoretical S-wave velocity and the black thin line is the initial S-wave velocity. SNR of the left column is 2.8 and SNR of the right column is 1.7. (**a**) Model 1; (**b**) Model 1; (**c**) Model 2; (**d**) Model 2.

We extract the dispersion curves of different components from the noisy synthetic seismic data and invert the S-wave velocity of Model 1 and Model 2, respectively, as shown in Figure 10:

The S-wave velocity inverted by the radial component is quite different from the theoretical model in the shallow layer, while that inverted by the vertical component has a great deviation from the real model in the deep layer. In contrast, the S-wave velocity inverted by the rotational component is the closest to the theoretical velocity. Reducing the signal-to-noise ratio has a minimal influence on the inversion results with rotation, while increasing the error of the results inverted with the translations.

## 6. Field Seismic Data Test

**Figure 11:**
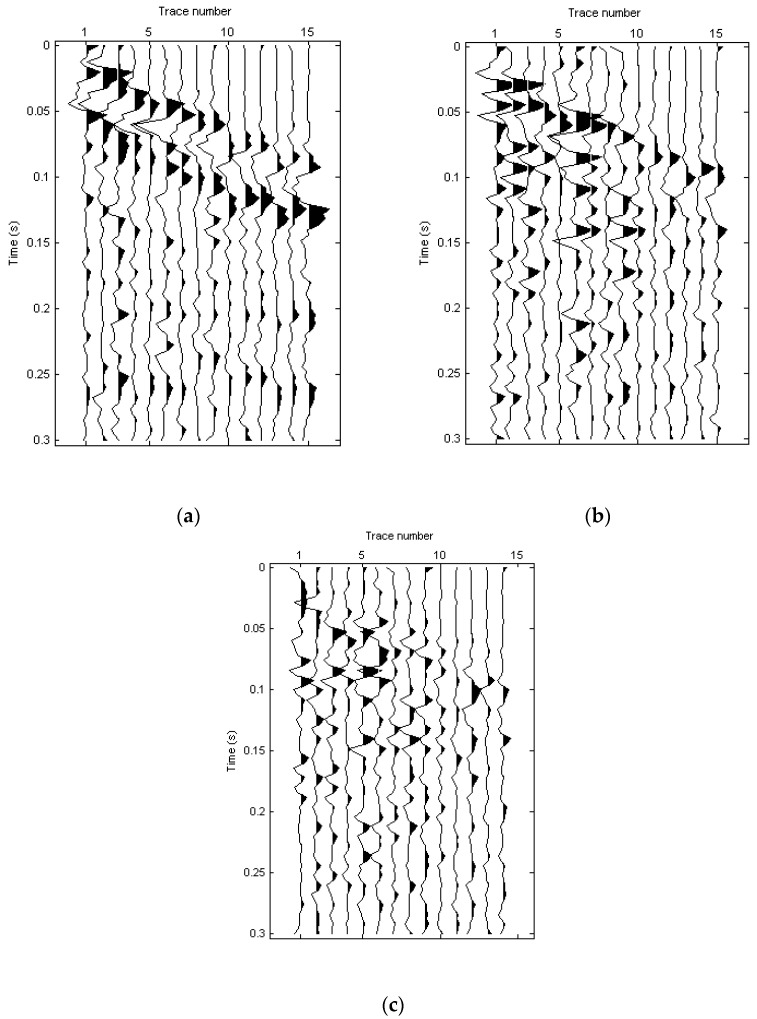
Field seismic data. (**a**) X; (**b**) Z; (**c**) R_y_.

Wanshousi Station, with a complex underground structure, is a key station of the Beijing No. 16 subway [50]. Beijing Petrosound Geoservices Stock Corp. was entrusted to carry out the two-dimensional three-component seismic observation and to detect the underground structure. They drop an iron hammer onto a solid fixture vertically to excite the seismic waves. There are 81 shots along one line and 15 three-component (3C) geophones with 1 m intervals. The nearest offset is 5 m and the time sample interval is 4 ms. Due to the lack of the rotational observation, we calculate the rotational component R_y_ with the two-point difference method. The translational and rotational components of the field data are illustrated in Figure 11.

**Figure 12:**
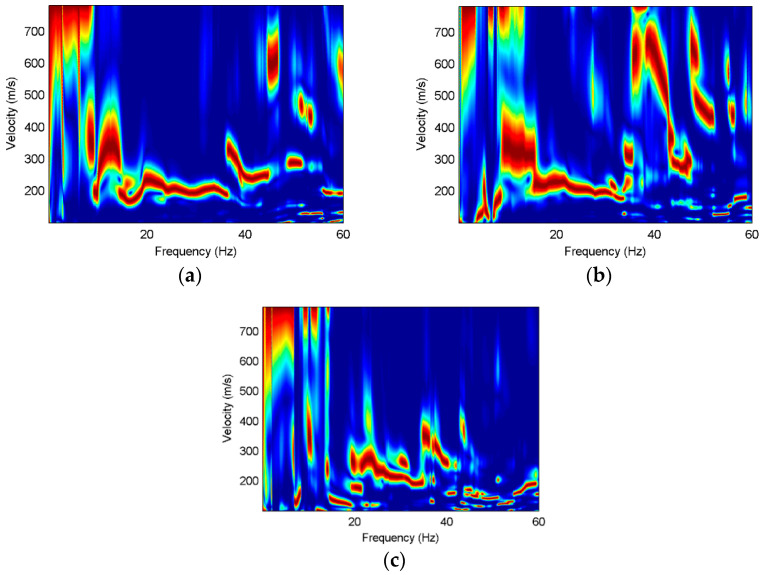
Normalized dispersion images of the field data, where the red color represents the maximum energy. (**a**) X; (**b**) Z; (**c**) R_y_.

It can be seen that there are obvious surface waves on the three components. The normalized dispersion images of three components are shown in Figure 12.

**Figure 13:**
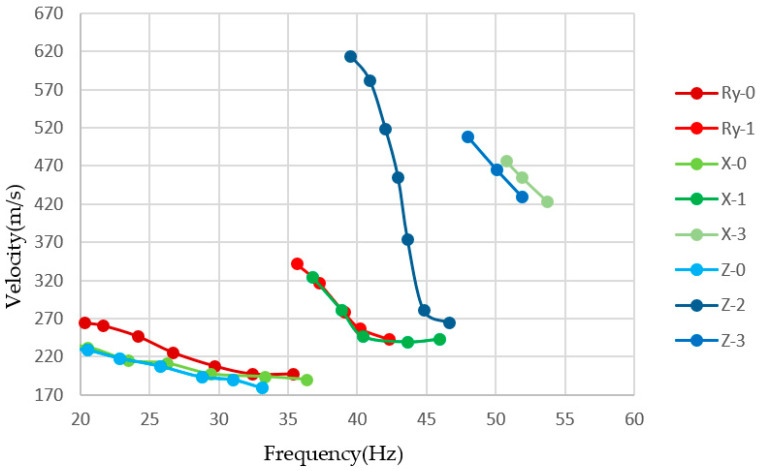
Comparison of dispersion curves extracted from different components.

It can be found that the fundamental mode has strong energy on the translational components, but not all higher modes are present on the three components. The first higher mode is dominant on the radial and rotational components, while the second higher mode and the third higher mode are obviously strong on the vertical component. The fundamental mode and the first higher mode on the rotational component have more low-frequency information while the radial component has richer dispersion information at high frequencies, as shown in Figure 13. Furthermore, the vertical component has higher dispersion modes. It can be demonstrated that the translation and rotation are complementary to each other, and more surface-wave dispersion information can be obtained by jointly using the three components.

**Figure 14:**
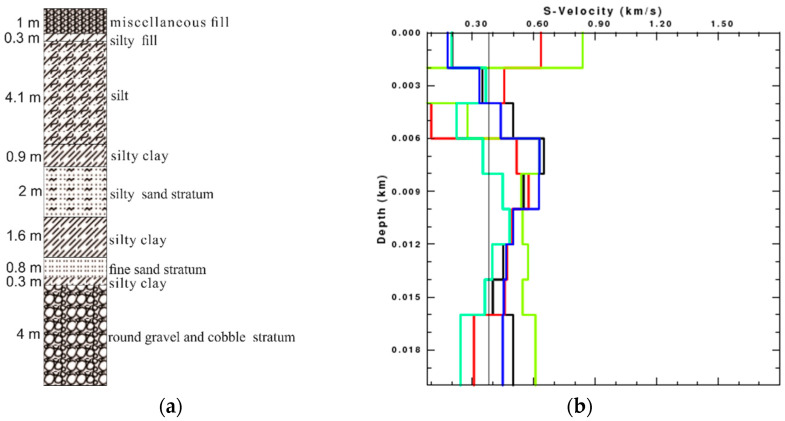
Field data test: (**a**) the lithologic histogram; (**b**) inversion results using the radial (red line), vertical (yellow line), rotational (green line), and multi-component (blue line) seismic data, respectively. The black thick line is the actual S-wave velocity, and the black thin line is the initial S-wave velocity.

There are three shafts in the construction area of Wanshousi station, and the lithologic histogram drawn according to the drilling core is shown in the Figure 14a. Based on the surface-wave dispersion curves in Figure 13, we invert the shallow S-wave velocity structure of 20 m underground by different components respectively, and then jointly use the translational and rotational components to obtain the underground S-wave velocity, as shown in Figure 14b. It can be found that the S-wave velocity inverted by the radial component has a great deviation in the shallow layer, which is the same as that inverted by the vertical component. The S-wave velocity inverted by the rotational component is slightly better than that inverted by the translational components, especially in the shallow layer. It is obvious that the S-wave velocity inverted by the rotational component has a smaller error than that inverted by the translational components, as shown in Table 4. The error of inversion results is calculated by:

(4) $$E=\sqrt{\frac{1}{n}\sum_{i=1}^{n}e_{i}^{2}}$$

where E is the error of inversion results, n is the number of the layers, and $e_{i}$ is the error of each layer, which is the deviation from the actual layer velocity.

The error of the S-wave velocity inverted by multi components is the smallest. The inversion results using the translational and rotational components jointly are close to the actual velocity, which shows the significant advantages of the multi-component joint inversion in the soft interlayer identification. It is demonstrated that the joint inversion of the translation and rotation can provide more accurate shear-wave velocity.

## 7. Discussion and Conclusions

We analyze the Rayleigh wave dispersion characteristics of translational and rotational components for three typical layered engineering models, compare the inversions of different components for synthetic noise-free and noisy data, and perform a test with a subway seismic prospecting case. The synthetic and field examples demonstrate the following:

1. The rotational component has more higher-mode dispersive energy and wider bandwidths than the translational components, particularly richer high-frequency information. The rotational and translational components supplement each other in terms of dispersion curves, which can provide more reliable dispersion information without mode misidentification.

2. The rotational component has stronger anti-noise capability than the translational components. Because there are wider frequency bands and more modes in noisy data, it can improve the inversion accuracy of the shallow shear-wave velocity.

3. The rotation can provide extra dispersion information in practical application. The joint utilization of the translational and rotational components has a considerable improvement on the weak layer identification and shows significant advantages in the shallow inversion.

It is obvious that the rotation is essential for a comprehensive description of the ground motion and that it can provide more accurate underground physical parameters by surface-wave inversion since the additional wave-field information can be obtained. Rotation is beneficial for shallow engineering exploration and surface-wave exploration.

There are still some deficiencies in this paper, since the rotational seismometers are not widely employed in the field observation, and there are non-ignorable differences between the array-derived and observed rotations, especially in the near-earthquake area [51,52]. However, in the future, it is worth popularizing the application of rotational seismometers in shallow seismic engineering and to identify high-precision surface-wave inversion by jointly using the translation and rotation.

## Figures and Tables

**Table 1:** The ground motion.

Axis	Translation		Rotation	
*x*	Radial	u_x_	Roll	r_x_
*y*	Transverse	u_y_	Pitch	r_y_
*z*	Vertical	u_z_	Yaw	r_z_

**Table 2:** The model parameters.

	Model 1			Model 2			Model 3		
Thickness	V_p_	V_s_	Den	V_p_	V_s_	Den	V_p_	V_s_	Den
5	600	200	1800	1100	300	1850	600	200	1800
5	1200	400	1900	600	200	1800	1800	800	2000
10	1800	800	2000	1800	800	2000	1300	600	1950
-	2900	1400	2100	2900	1400	2100	2900	1400	2100

Thickness, (m); V_p_, the velocity of P-waves (m/s); V_s_, the velocity of S-waves (m/s); Den, density (kg/m^3^).

**Table 3:** Comparison of bandwidths among the radial, vertical and rotational data.

	Component	Fundamental Mode (Hz)	First Higher Mode (Hz)	Second Higher Mode (Hz)	Third Higher Mode (Hz)
Model 1	radial	10–15, 18–100	12–15	-	50–58
	vertical	10–100	44–50	68–72	-
	rotational	10–15, 18–100	12–15, 22–42	32–42, 60–84	48–86, 92–100
Model 2	radial	8–32	32–54	44–75	30–36
	vertical	8–32	26–54	36–75	-
	rotational	8–32	22–28, 32–54	44–75	28–52

**Table 4:** Error of the inversion results using the radial (X), vertical (Z), rotational (R_y_), and multi-component (X + Z + R_y_) seismic data.

	Depth (m)	X	Z	R_y_	X + Z + R_y_
$e_{i}$	0~2	0.435	0.638	−0.001	−0.021
	2~4	0.105	−0.301	0.016	0.005
	4~6	−0.401	−0.224	−0.278	−0.061
	6~8	−0.135	−0.022	−0.299	−0.023
	8~10	0.024	−0.012	−0.103	0.075
	10~12	−0.005	0.045	−0.019	−0.002
	12~14	0.020	0.122	−0.053	0.016
	14~16	0.058	0.146	−0.040	0.053
	-	−0.193	0.108	−0.257	−0.052
E	-	0.205	0.245	0.158	0.040

## Data Availability

Not applicable.
